# Extrapulmonary Nontuberculous Mycobacteria Infections in Hospitalized Patients, United States, 2009–2014

**DOI:** 10.3201/eid2703.201087

**Published:** 2021-03

**Authors:** Emily E. Ricotta, Jennifer Adjemian, Rebekah A. Blakney, Yi Ling Lai, Sameer S. Kadri, D. Rebecca Prevots

**Affiliations:** National Institutes of Health National Institute of Allergy and Infectious Diseases, Bethesda, Maryland, USA (E.E. Ricotta, J. Adjemian, R.A. Blakney, Y.L. Lai, D.R. Prevots);; US Public Health Service Commissioned Corps, Rockville, Maryland, USA (J. Adjemian);; National Institutes of Health Clinical Center, Bethesda (S.S. Kadri)

**Keywords:** Epidemiology, nontuberculous mycobacteria, electronic health record, tuberculosis and other mycobacteria, United States, bacteria

## Abstract

Nontuberculous mycobacteria (NTM) cause pulmonary and extrapulmonary infections in susceptible persons. To characterize the epidemiology of skin and soft tissue (SST) and disseminated extrapulmonary infections caused by NTM in the United States, we used a large electronic health record database to examine clinical, demographic, and laboratory data for hospitalized patients with NTM isolated from extrapulmonary sources during 2009–2014. Using all unique inpatients as the denominator, we estimated prevalence and summarized cases by key characteristics. Of 9,196,147 inpatients, 831 had confirmed extrapulmonary NTM. The 6-year prevalence was 11 cases/100,000 inpatients; source-specific prevalence was 4.4 SST infections/100,000 inpatients and 3.7 disseminated infections/100,000 inpatients. NTM species varied across geographic region; rapidly growing NTM were most prevalent in southern states. Infection with *Mycobacterium avium* complex was more common among patients with concurrent HIV and fungal infection, a relevant finding because treatment is more effective for *M. avium* complex than for other NTM infections.

Nontuberculous mycobacteria (NTM) are opportunistic bacteria that are abundant in soil and water, including natural and plumbing-associated water sources (*1*,*2*). For a minority of susceptible persons, exposure to NTM can result in extrapulmonary infections (*3*), including skin, joint, lymph node, and disseminated infections. Extrapulmonary infections, especially disseminated disease, typically occur among persons with congenital or acquired immunodeficiencies (e.g., HIV infection) (*4*) but can also be associated with medical or cosmetic procedures that expose a wound to sources contaminated with mycobacteria (*5*,*6*). A recently described outbreak identified disseminated infections with *Mycobacterium chimaera* after open heart surgery, arising from contamination of heater–cooler units (*6*).

Few studies describe the epidemiology of extrapulmonary NTM in the United States at the national level. One recent study in Oregon evaluated the prevalence of extrapulmonary NTM by using statewide population-based laboratory surveillance data for 2007–2012, which included data for pulmonary and extrapulmonary NTM (*4*). The researchers estimated a stable annual incidence of extrapulmonary NTM infection of 1.5 cases/100,000 population. The average age of extrapulmonary NTM patients (median 51 years) was younger than that of pulmonary NTM patients. In addition, rapidly growing NTM species were identified at a much greater frequency in extrapulmonary than in pulmonary NTM patients and represented one third of all cases in Oregon (*4*). Epidemiologic studies of pulmonary NTM disease show tremendous geographic variation in prevalence and mycobacterial species (*7*,*8*), suggesting the possibility of differences for extrapulmonary NTM as well, given the environmental influences on NTM disease dynamics. To characterize the epidemiology of skin and soft tissue (SST) and disseminated NTM infections and evaluate regional differences in incidence and mycobacterial species distribution, we examined laboratory-confirmed cases from a large electronic health record (EHR)–based repository of inpatient encounters from a national sample of US hospitals. 

## Methods

The nationally distributed, hospital-based Cerner Health Facts EHR database (https://sc-ctsi.org/resources/cerner-health-facts) includes linked demographic, clinical, and microbiological information for ≈9 million US inpatients. Using this database, we identified all US patients hospitalized during 2009–2014 with positive NTM cultures from extrapulmonary sources (excluding *M. gordonae* because it is considered an environmental contaminant) (Appendix Table 1). Patients were classified as having SST disease, disseminated disease (including those with infections in blood, central nervous system, and sterile bone and joint sources), or both; patients with infections from abdominal sites, urinary system, or other body sites were also identified and grouped as other sources (Table 1; Appendix Tables 2, 3). Sources were further classified as sterile or not sterile and whether they were associated with a device, prosthesis, or surgical procedure (Table 2). We excluded from analysis 142 patients with isolates from unknown sources and 4,385 patients with isolates from pulmonary sources.

**Table 1:** Classification of extrapulmonary nontuberculous mycobacterial infection type, by body site, United States, 2009–2014*

Infection type	Site
Skin and soft tissue	Arm, boil, cheek, ear, foot, genital, groin, incision, leg, lymph node, mass, neck, node, nodule, skin, thigh, tissue, wound
Disseminated	
Blood	Blood, blood capillary, blood line, blood venous, blood whole, central line
Bone and joint (sterile)	Bone, bone marrow, wrist, synovial fluid, jaw, joint fluid, knee, hip
Central nervous system	Cerebrospinal fluid
Other	
Abdominal	Liver, ascites fluid, gastric tube, abdominal fluid, gastric fluid, nasogastric aspirate, peritoneal, peritoneal dialysis fluid, peritoneal fluid, gastric aspirate, perianal, colonic wash, feces, rectal, percutaneous endoscopic gastrostomy site
Urinary	Urine, urine catheterized, urine clean catch, urine midstream, urine voided
Other	Eye fluid, cervical, pericardial fluid, sternal, exit site, foreign body, pacemaker, plate, prosthesis, surgical, nasopharynx, throat, nonsterile bone and joint

*Data from in Cerner Health Facts database (https://sc-ctsi.org/resources/cerner-health-facts).

**Table 2:** Sources of extrapulmonary nontuberculous mycobacterial infection, by site sterility and association with medical device, prosthetics, and surgery, United States, 2009–2014*

Source	Sterile, no. (%)	Not sterile, no. (%)	Device/prosthesis associated, no. (%)	Surgery-associated, no. (%)
Skin and soft tissue, n = 340	33 (10)	307 (90)	59 (17)	129 (38)
Disseminated, n = 290				
Blood, n = 259	259 (100)	NA	72 (28)	26 (10)
Bone and joint, n = 26	26 (100)	NA	7 (27)	7 (27)
Central nervous system, n = 5	5 (100)	NA	3 (60)	3 (60)
Other, n = 362				
Abdominal, n = 110	21 (19)	89 (81)	27 (24)	17 (15)
Urinary, n = 11	0	11 (100)	2 (18)	1 (9)
Other, n = 241	64 (27)	177 (73)	49 (20)	87 (36)

*Data from in Cerner Health Facts database (https://sc-ctsi.org/resources/cerner-health-facts). NA, not applicable.

**Table 3:** Nontuberculous mycobacteria species distribution overall and by source, United States, 2009–2014*

Species	Total no. (%)	Disseminated, no. (%)	Skin and soft tissue, no. (%)	Other, no. (%)
*Mycobacterium avium* complex	501 (50)	157 (54)	177 (52)	167 (45)
*M. abscessus*	94 (9)	24 (8)	27 (8)	43 (12)
*M. abscessus/chelonae*	43 (4)	13 (4)	15 (4)	15 (4)
*M. chelonae*	53 (5)	14 (5)	21 (6)	18 (5)
*M. fortuitum*	104 (10)	20 (7)	36 (11)	48 (13)
*M. kansasii*	26 (3)	6 (2)	9 (3)	11 (3)
*Mycobacterium* spp.	79 (8)	17 (6)	20 (6)	42 (11)
Other non–rapidly growing NTM	37 (4)	3 (1)	20 (6)	14 (4)
Other rapidly growing NTM	61 (6)	36 (12)	15 (4)	10 (3)
Total	998 (100)	290 (29)	340 (34)	368 (37)

*Data from in Cerner Health Facts database (https://sc-ctsi.org/resources/cerner-health-facts).

Patients with extrapulmonary NTM were described by demographic factors (age, sex, race, and geographic region) and clinical factors (underlying conditions and procedural history via codes from the International Classification of Diseases, Ninth and Tenth Revisions, and Current Procedural Terminology). To compare demographics by infection type, we used the Pearson χ^2^ test or analysis of variance, where appropriate. We calculated overall and annual inpatient prevalence estimates by determining the number of unique inpatients with >1 positive extrapulmonary NTM culture divided by the total number of unique inpatients identified during the study period among hospitals reporting >1 case of extrapulmonary NTM. Patients whose cultures grew multiple NTM species or had isolates cultured from multiple extrapulmonary sites were counted in each group unless specified. Statistical analyses were conducted by using R version 4.0.2 (https://www.R-project.org).

## Results

Of 9,196,147 unique inpatients from 275 inpatient facilities reporting culture results throughout the United States, laboratory-confirmed extrapulmonary NTM was reported for 998 unique species/source isolates from 831 patients at 89 hospitals. Isolates represented 321 (39%) patients with SST infections, 269 (32%) with disseminated infections, and 337 (41%) with infection at other sites. Both disseminated and SST infections were reported for 23 (2.8%) patients. Most isolates identified to the species level were *Mycobacterium avium* complex (MAC) (50%), followed by *M. fortuitum* (10%), *M. abscessus* (9.4%), *M. chelonae* (5.3%), and *M. chelonae/abscessus* (4.3%). Other species were rapidly growing NTM (8.7%), non–rapidly growing NTM (3.7%), or not speciated (7.9%) (Table 3).

**Figure 1:**
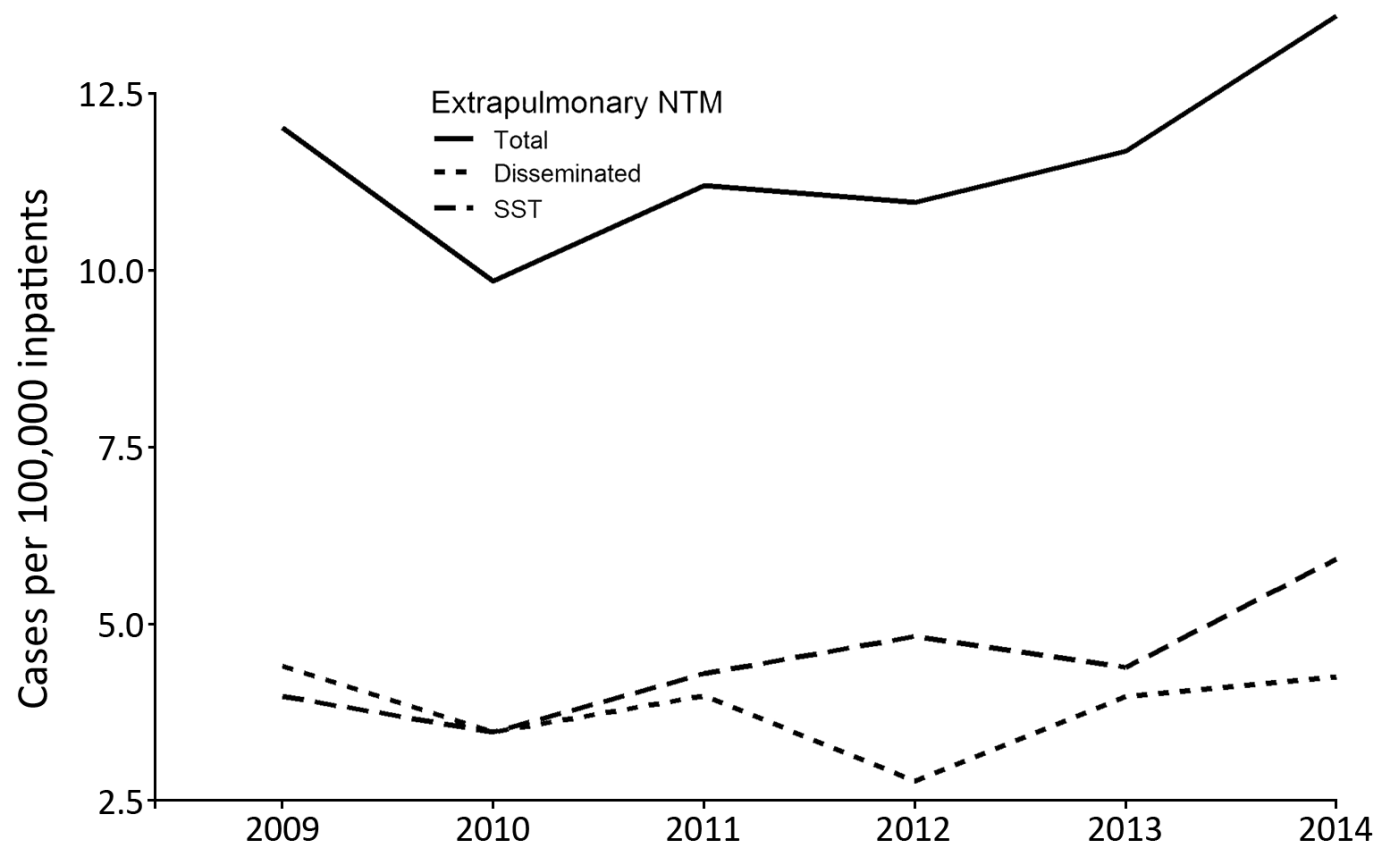
Annual prevalence of extrapulmonary nontuberculous mycobacteria cases by year and site of infection among hospitalized patients in the United States, 2009–2014. SST, skin and soft tissue.

The overall 6-year prevalence of extrapulmonary NTM in hospitals reporting >1 inpatient with extrapulmonary NTM was 11 cases/100,000 inpatients. Site-specific infections were 4.4 SST infections/100,000 inpatients, 3.7 disseminated infections/100,000 inpatients, and 0.3 cases of both types of infection/100,000 inpatients. Annual prevalence of disseminated NTM remained stable over the study period, whereas SST infections increased 8.2% (95% CI 1%–15%) (Figure 1). Prevalence was highest in the Midwest (13 cases/100,000 inpatients), South (13 cases/100,000 inpatients), and Northeast (11 cases/100,000 inpatients) and lowest in the West (5.3 cases/100,000 inpatients).

Among patients, 49% were female, 58% were White, and 60% were >40 years of age; 32% were Black and 11% were <18 years of age. Relative to patients with SST infections, those with disseminated cases were more frequently male (60% vs. 45%; p<0.001), younger (mean age 40 vs. 50 years; p<0.001), and Black (56% vs. 13%; p<0.001). Among patients with both SST and disseminated infection, 61% were female, most (52%) were White, and mean age was 52 years (Table 4). Among patients with SST infections, 15% had undergone a surgical procedure (e.g., invasive, minimally invasive, surgical biopsy) compared with 4% of patients with disseminated infection. Among all patients, 20% had ever taken an immunosuppressive drug (Table 4); among these, 19% had SST infection, 23% had disseminated infection, and 22% had both. Crude overall mortality rate was 5% (11% among those with disseminated and 2% among those with SST infections); 1 patient with both types of infection died.

**Table 4:** Demographic and clinical characteristics of extrapulmonary nontuberculous mycobacteria cases among hospitalized patients from 82 hospitals, United States, 2009–2014*

Characteristics	Extrapulmonary NTM, no. (%), n = 831	Disseminated, no. (%), n = 246	SST, no. (%), n = 298	Other, no. (%), n = 264	Both, no. (%), n = 23
Patient characteristic					
Sex					
F	409 (49)	98 (40)	164 (55)	133 (50)	14 (61)
M	422 (51)	148 (60)	134 (45)	131 (50)	9 (39)
Race/ethnicity					
White	478 (58)	93 (38)	228 (77)	145 (55)	12 (52)
Black	269 (32)	138 (56)	38 (13)	83 (31)	10 (43)
Other	84 (10)	15 (6)	32 (11)	36 (14)	1 (4)
Age group, y					
<18	91 (11)	13 (5)	43 (14)	35 (13)	0
>18–40	244 (29)	121 (49)	47 (16)	70 (27)	6 (26)
>40–60	266 (32)	86 (35)	93 (31)	78 (30)	9 (39)
>60	230 (28)	26 (11)	115 (39)	81 (31)	8 (35)
Ever had					
Fungal Infection	92 (11)	53 (22)	13 (4)	22 (8)	4 (17)
HIV infection	104 (13)	63 (26)	9 (3)	26 (10)	6 (26)
Invasive cardiac procedure	18 (2)	4 (2)	6 (2)	7 (3)	1 (4)
Cancer	31 (4)	11 (4)	9 (3)	11 (4)	0
Other immunologic disorder†	18 (2)	8 (3)	3 (1)	7 (3)	0
In-hospital death or discharged to hospice	47 (6)	29 (12)	5 (2)	12 (5)	1 (4)
Hospital characteristic					
Region					
South	375 (45)	147 (60)	94 (32)	125 (47)	9 (39)
Northeast	202 (24)	44 (18)	95 (32)	56 (21)	7 (30)
Midwest	200 (24)	44 (18)	88 (30)	64 (24)	4 (17)
West	54 (6)	11 (4)	21 (7)	19 (7)	3 (13)
Setting					
Urban	764 (92)	231 (94)	278 (93)	234 (89)	21 (91)
Rural	67 (8)	15 (6)	20 (7)	30 (11)	2 (9)
Teaching status‡					
Teaching facility	686 (83)	210(85)	247 (83)	209 (79)	20 (87)
Not teaching facility	112 (13)	30 (12)	35 (12)	209 (17)	3 (13)

*Data from in Cerner Health Facts database (https://sc-ctsi.org/resources/cerner-health-facts). Both, disseminated and SST infection; SST, skin and soft tissue.  †Antineoplastic and immunosuppressive drugs causing adverse effects in therapeutic use, autoimmune disease, not elsewhere classified, common variable immunodeficiency, encounter for antineoplastic immunotherapy, immunodeficiency with predominant T-cell defect, unspecified, other and unspecified nonspecific immunological findings, other specified disorders involving the immune mechanism, personal history of immunosuppression therapy, unspecified disorder of immune mechanism, unspecified immunity deficiency. ‡Status unknown for 33 patients.

**Figure 2:**
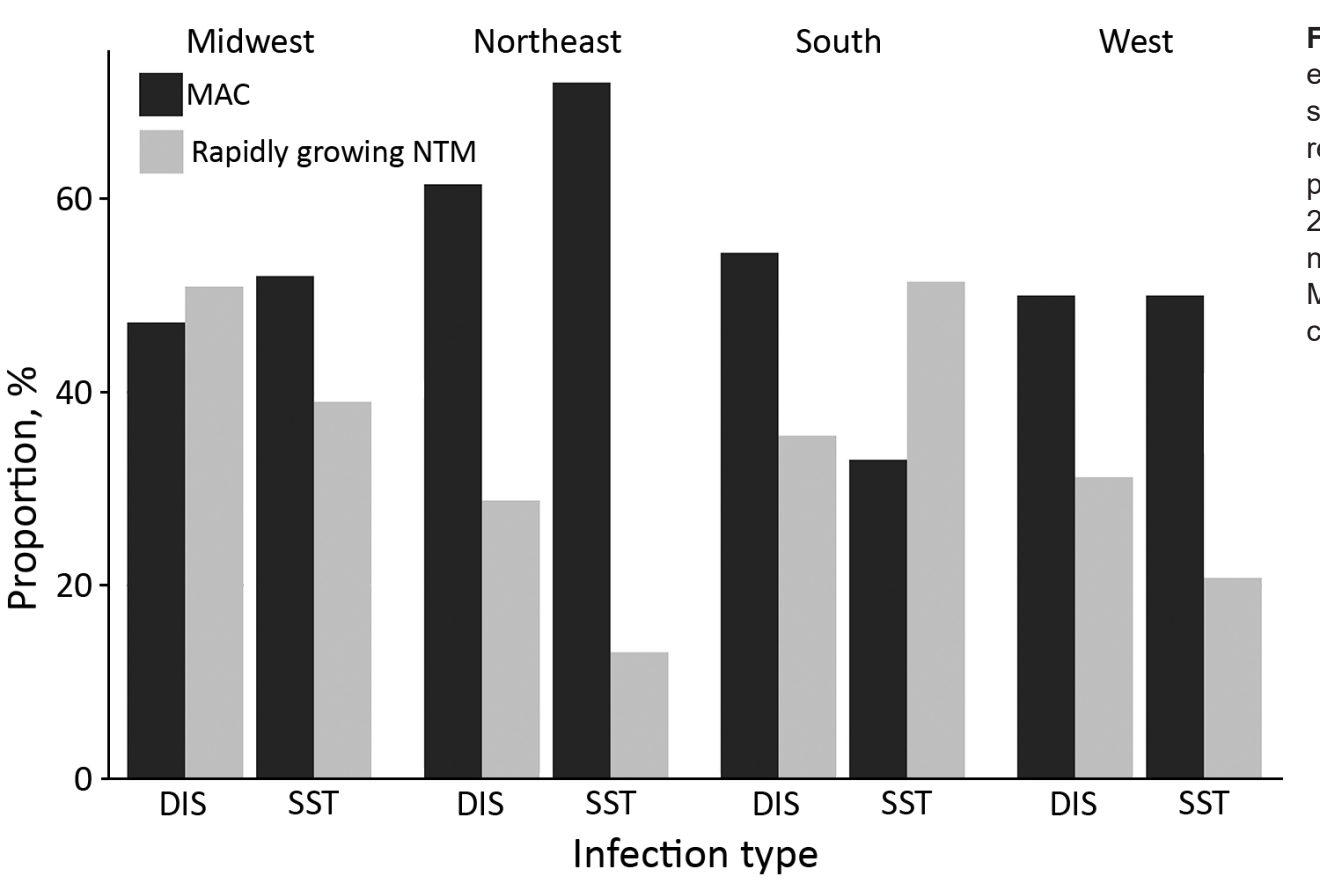
Distribution of extrapulmonary NTM cases by species and infection type across regions among hospitalized patients in the United States, 2009–2014. DIS, disseminated; NTM, nontuberculous mycobacteria; MAC, *Mycobacterium avium* complex; SST, skin and soft tissue.

MAC accounted for more than half of disseminated (54%) and SST infections (52%), and rapidly growing NTM accounted for 34% of SST infections and 37% of disseminated infections. Distribution of cases by source and species varied by region (Table 4). SST infections were more common in the Midwest (30% vs. 18%; p = 0.002) and Northeast (32% vs. 18%; p<0.001), and disseminated infections were more common in the South (60% vs. 32%; p<0.001). MAC was found at a higher proportion than rapidly growing NTM in the Northeast (30% vs. 13%; p<0.001), and rapidly growing NTM were found at a higher proportion in the Midwest (32% vs. 23%; p = 0.004) and South (52% vs .40%; p = 0.001). When infections were broken down further by species and infection type, a significantly higher proportion of MAC was found in the Northeast for disseminated (62% vs. 29%; p = 0.002) and SST infections (72% vs. 12%; p<0.001) and in the South for disseminated infections (54% vs. 36%; p<0.001). Compared with MAC, the proportion of rapidly growing NTM causing SST infections was higher in the South (51% vs. 33%; p = 0.009) (Figure 2).

Underlying conditions included fungal co-infections (11%), HIV infection (13%), cancer (4%), and other immunodeficiencies (2%); 14 (2%) NTM patients had a history of invasive cardiac procedures (Table 4). A higher proportion of patients with MAC than with rapidly growing NTM had HIV infection (21% vs. 1.5%; p<0.001) and fungal infections (16% vs. 6.7%; p<0.001), and a higher proportion of patients with rapidly growing NTM had cancer (6.7% vs. 1.4%; p<0.001). Co-infections (including pulmonary pathogens) identified during the same hospitalization as NTM isolation were common; >1 concomitant pathogen of interest grew for 42% of patients (Appendix Table 4). 

**Figure 3:**
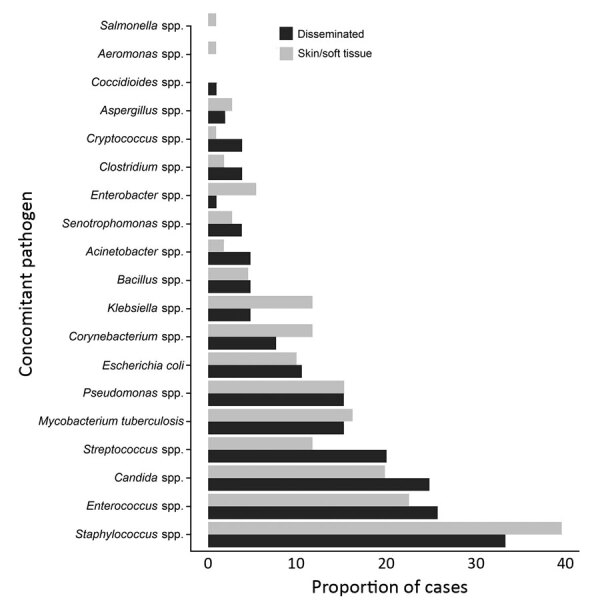
Distribution of laboratory-confirmed concomitant pathogens by infection type among hospitalized patients with extrapulmonary nontuberculous mycobacteria, United States, 2009–2014.

By extrapulmonary NTM infection type, co-infection was found for 37% of patients with SST, 47% with disseminated, and 61% with both. Among all persons with co-infection, 13% had *Staphylococcus* spp., 10% had *Candida* spp., 9.0% had *Enterococcus* spp., 7.0% had *Streptococcus* spp., 6.6% had *Pseudomonas* spp., 5.2% had *Escherichia coli,* 3.8% had *Klebsiella* spp., and 8.2% had *M. tuberculosis* complex (MTBC) (Table 5). Of patients with both SST and disseminated NTM, 36% were co-infected with *Enterococcus* spp., 26% with MTBC, and 29% with *Staphylococcus* spp. (Figure 3). Patients with disseminated NTM had a higher proportion of *Acinetobacter* spp., *Bacillus* spp., *Candida* spp., *Clostridium* spp., *Coccidioides* spp., *Cryptococcus* spp., *Enterococcus* spp., *E. coli*, *Stenotrophomonas* spp., and *Streptococcus* spp. infection; patients with SST NTM infection had a higher proportion of *Aeromonas* spp., *Aspergillus* spp., *Corynebacterium* spp., *Enterobacter* spp., *Klebsiella* spp., MTBC, *Salmonella* spp., and *Staphylococcus* spp., although the differences were not significant (Table 5; Figure 3).

**Table 5:** Concomitant organisms isolated from hospitalized patients with extrapulmonary nontuberculous mycobacteria, overall and by source, United States, 2009–2014*

Genus	Total, no. (%)	Disseminated, no. (%)	SST, no. (%)	Both, no. (%)	Other, no. (%)
*Acinetobacter*	9 (3)	5 (5)	2 (2)	0	2 (2)
*Aeromonas*	3 (0.9)	0	1 (1)	0	2 (2)
*Aspergillus*	7 (2)	2 (2)	3 (3)	0	2 (2)
*Bacillus*	15 (4)	5 (5)	5 (5)	1 (7)	4 (3)
*Candida*	80 (23)	26 (25)	22 (20)	1 (7)	31 (26)
*Clostridium*	9 (3)	4 (4)	2 (2)	0	3 (3)
*Coccidioides*	2 (0.6)	1 (1)	0	0	1 (1)
*Corynebacterium*	31 (9)	8 (8)	13 (12)	0	10 (8)
*Cryptococcus*	6 (2)	4 (4)	1 (1)	0	1 (1)
*Enterobacter*	13 (4)	1 (1)	6 (5)	0	6 (5)
*Enterococcus*	75 (21)	27 (26)	25 (23)	5 (36)	18 (15)
*Escherichia coli*	43 (12)	11 (11)	11 (10)	1 (7)	20 (17)
*Klebsiella*	32 (9)	5 (5)	13 (12)	1 (7)	13 (11)
*Mycobacterium tuberculosis*	68 (19)	16 (15)	18 (16)	5 (36)	29 (24)
*Pseudomonas*	55 (16)	16 (15)	17 (15)	0	22 (18)
*Salmonella*	2 (0.6)	0	1 (1)	0	1 (1)
*Staphylococcus*	112 (32)	35 (33)	44 (40)	4 (29)	29 (24)
*Stenotrophomonas*	11 (3)	4 (4)	3 (3)	1 (7)	3 (3)
*Streptococcus*	58 (17)	21 (20)	13 (12)	0	24 (20)
Total patients with co-infection	350	105 (30)	111 (32)	14 (4)	120 (34)

*Data from in Cerner Health Facts database (https://sc-ctsi.org/resources/cerner-health-facts). Both, disseminated and SST infection; SST, skin and soft tissue.

## Discussion

Using the Cerner Health Facts EHR database, we found that the annual prevalence of extrapulmonary NTM overall was stable over time and that SST NTM infections increased significantly, which could result from the increased number of patients taking immunosuppressive drugs (20% of patients in this cohort) or increased cosmetic procedures (e.g., tattooing, pedicures) (*10*,*11*). Although population-based studies have found a lower and stable prevalence of extrapulmonary NTM, it is possible that the higher prevalence we found results from patients having more severe infections that necessitate testing, increasing the chances of diagnosing this disease (*4*,*11*).

NTM infections varied by geographic region in prevalence, infection type, and mycobacterial species. Specifically, prevalence of extrapulmonary NTM was higher among hospitalized patients in the South, Midwest, and Northeast than in the West, although these high rates resulted from disseminated infection in the South versus a more even distribution of SST infections in other regions. Recent studies of extrapulmonary NTM in the United States have focused on specific geographic locations. In Oregon, Shih et al. (*12*) and Henkle et al. (*4*) analyzed all extrapulmonary NTM cases identified via statewide laboratory-based active surveillance efforts and estimated incidence rates to be 1.1–1.5 cases/100,000 persons/year, with only one third of those patients hospitalized (*12*). These estimates are substantially lower than those reported in North Carolina (*13*), where a similar surveillance-based study estimated the prevalence among residents of 3 counties to be ≈3 cases/100,000 persons. The differences in prevalence estimates between Oregon and North Carolina similarly reflect the regional differences that we observed; prevalence was higher in southern than in western states. The geographic variations in prevalence of extrapulmonary NTM cases that we found are also similar to what has been shown in US population–level pulmonary NTM studies (*8*,*14*,*15*), that residents of southern states are at increased risk for NTM lung disease, particularly among high-risk groups such as persons with cystic fibrosis (*15*–*17*). Differences by geographic region are largely associated with environmental factors, such as greater amounts of water on land and in the lower level atmosphere (*14**–**16*), which probably contributes to increased environmental abundance of mycobacteria. In addition to higher levels of exposure to mycobacteria, studies have identified that these high-risk areas also tend to have a higher proportion of rapidly growing NTM species relative to MAC or other mycobacteria (*7*,*15*,*17*), which can result in more severe disease with limited effective treatment options (*3*).

Among extrapulmonary NTM cases, mycobacteria species also varied greatly by infection source and underlying condition. Although MAC infections were most frequent across all types of extrapulmonary NTM cases, in certain regions rapidly growing NTM play a substantial role in causing disease. Nearly all patients with HIV had MAC; those with a history of cancer were more likely to have rapidly growing NTM. Given that species of rapidly growing NTM, particularly *M. abscessus* and *M. fortuitum*, which were the most prevalent species in this study, are typically more challenging to treat than MAC, these findings have implications for the clinical management of these patients with complex infections and medical conditions. Co-infections were common among patients with extrapulmonary NTM, and >1 other pathogen was isolated from nearly half of all patients. Co-infections may complicate treatment-related decisions, particularly if mycobacteria, which are typically slow growing, are detected after other pathogens and are not treated with appropriate antimicrobial drug therapy.

Because we evaluated ≈9 million unique persons from 275 hospitals across the United States, we were able to identify key epidemiologic patterns for what is otherwise a very rare disease with limited population-level data. Because our analysis included only hospitalized patients, we probably overestimated the true incidence of extrapulmonary NTM disease in the general population by selecting for generally sicker patients with more severe underlying disease. We may have missed less severe SST infections that did not require extensive treatment or hospital intervention. Because the hospitals included here represent only those that use the Cerner Health Facts system, this study does not include patients at other facilities, which may also affect our incidence calculations. Similarly, not captured here were surgeries, procedures, or prior medical events that occurred in other facilities, which may be associated with risk, infection type, and outcome. However, these limitations would be applied systematically to the entire study population and therefore would probably not alter the geographic or temporal patterns that we found.

Overall, extrapulmonary NTM disease remains rare with relatively stable incidence rates for disseminated NTM infections and modestly increased rates for SST infections. In similar studies assessing pulmonary NTM, rates appear to be steadily increasing in the general population and among high-risk groups such as persons with cystic fibrosis (*8*,*17*). Patients with extrapulmonary NTM typically include persons with HIV, other underlying immunodeficiencies, histories of surgical procedures, or other unique exposures that increase the risk for infection. In addition, we found that species variability is associated with geographic region; rapidly growing NTM are more prevalent in the southern United States than in other regions. Given the added treatment challenges that exist for these patients with often-complex conditions, knowledge of key trends and risks by patient-level factors and geographic location is critical for improving clinical outcomes and determining sources of infections that may be common to patients with pulmonary and extrapulmonary NTM.

Appendix Additional methods and results for study of extrapulmonary nontuberculous mycobacterial infections in hospitalized patients, United States, 2009–2014.
