# Supplementary material for: Extrapulmonary Nontuberculous Mycobacteria Infections in Hospitalized Patients, United States, 2009–2014
Source: Emerg Infect Dis. 2021 Mar;27(3):845–52. doi: 10.3201/eid2703.201087 (PMC7920686; doi:10.3201/eid2703.201087)
Supplement: Appendix — Additional methods and results for study of extrapulmonary nontuberculous mycobacterial infections in hospitalized patients, United States, 2009–2014. [file 20-1087-Techapp-s1.pdf]

Article DOI: https://doi.org/10.3201/eid2703.201087

Extrapulmonary Nontuberculous Mycobacterial Infections in Hospitalized Patients, United States, 2009–2014

Appendix

Appendix Table 1. Hospital characteristics of Cerner *HealthFacts* hospitals reporting extrapulmonary nontuberculous mycobacteria compared to all inpatient facilities, and the American Hospital Association (AHA) Acute Care Hospitals, 2014[1]

	Study hospitals		All Cerner inpatient facilities 2009-2014		AHA hospitals	
Hospital characteristic	N=89	% of study cohort	N=378	% of hospitals	N=4810	% of AHA hospitals
Geographic region						
Midwest	20	22	76	20	1096	23
Northeast	25	28	72	19	598	12
South	32	36	128	34	2168	45
West	12	13	102	27	948	20
Teaching status						
Teaching	52	58	111	29	1425	30
Nonteaching	32	36	241	64	3385	70
Unknown	5	6	26	7	N/A	N/A
AHA hospital size*						
Small (<200)	27	30	270	71	2606	54
Medium (200-499)	52	58	84	22	1939	40
Large (≥500)	10	11	23	6	265	6

*Hospital size for 1 Cerner facility was unknown

Appendix Table 2. Breakdown of collection sources and sites for extrapulmonary nontuberculous mycobacterial infections in hospitalized patients, United States, 2009-2014.

Collection Site	Collection source
**Sterile SST**	
Abdominal	Lymph Node
Femur	Mass
Groin	Tissue
Heart	Wound
Liver	
Lymph node	
Neck	
Pericardial fluid	
Shoulder	
Spleen	
Subclavian catheter	
Unknown	
**Nonsterile SST**	
**Collection site**	**Collection source**
Abdominal	Arm
Abscess	Boil
Ankle	Chest
Arm	Ear
Axilla	Foot
Back	Hip
Bone	Incision
Breast	Knee
Buttock	Leg
Calf	Mass
Cervical	Neck
Chest	Node
Ear	Other
Elbow	Skin
Face	Sternal

Collection Site	Collection source
Fine needle aspirate	Swab
Finger	Thigh
Foot	Thumb
Forearm	Tissue
Groin	Unknown
Hand	Wound
Head	Wrist
Hip	
Humerus	
Ileum	
Knee	
Leg	
Lesion	
Lumbar	
Lymph node	
Mass	
Muscle	
Neck	
Other	
Pharynx	
Recipient	
Rib	
Sacral	
Scalp	
Shoulder	
Spinal	
Sternal	
Submandibular gland	
Thigh	
Thumb	
Tibia	
Tissue	
Toe	
Vulva	
Wound	
Wrist	
Unknown	
**Disseminated**	
Ankle	Blood
Antecubital	Blood Capillary
Arm	Blood Line
Blood	Blood Peripheral
Blood peripheral	Blood venous
Blood venous	Blood whole
Bone	Bone
Bone marrow	Bone marrow
Catheter	Central Line
Central line	Cerebrospinal fluid
Cerebrospinal fluid	Joint fluid
Elbow	Other
Femur	Synovial Fluid
Foot	Unknown
Forearm	
Hand	
Hip	
Iliac crest	
Knee	
Leg	
Line blood	
Lumbar	
Lumen	
Other	
Picc line	
Port	
Port-a-cath	
Subclavian	
Vertebra	
Wrist	
Unknown	

Appendix Table 3. Microbiology procedures isolating extrapulmonary nontuberculous mycobacteria, hospitalized patients, United States, 2009-2014

Lab procedure names
Aerobic Culture Bacteria Identified Unspecified
Aerobic/Anaerobic Culture, Wound
AFB Culture
AFB Culture Abcess
AFB Culture and Smear/Stain
AFB Culture Blood
AFB Culture CSF
AFB Culture Tissue
Anaerobe+Aerobe Bacteria Identified Unspecified Culture
Anaerobic Culture Bacteria Identified Unspecified
Bartonella Identified Unspecified Organism Specific Culture
Blood Culture
Blood Culture AFB
Blood Culture ARD
Blood Culture Fungus
Culture Abcess
Culture and Gram Stain Aerobic/Anaerobic
Culture and Gram Stain CSF
Culture and Gram Stain Tissue
Culture and Gram Stain Wound
Culture Biopsy
Culture Ear
Culture Miscellaneous
Culture Routine
Culture Tissue
Culture Wound
Fungal Culture and Stain
Fungal Culture Bone Marrow
Fungal Culture Tissue
Fungal Identified Unspecified Culture
Unknown

Appendix Table 4. Distribution of coinfections by body source for hospitalized patients with extrapulmonary nontuberculous mycobacteria, United States, 2009–2014*

Coinfection	Blood	CNS	Sterile bone/join	Not sterile bone/joint	SST	Not sterile SST	Sterile abdominal	Not sterile abdominal	Other sterile	Not sterile other	Pulmonary	Urine
Acinetobacter	6 (67)	0	1 (11)	0	0	1 (11)	0	0	0	1 (11)	0	0
Aeromonas	0	0	0	0	0	2 (67)	0	0	0	1 (33)	0	0
Aspergillus	2 (25)	0	0	0	0	2 (25)	0	0	0	2 (25)	2 (25)	0
Bacillus	5 (33)	0	1 (7)	0	0	4 (27)	0	1 (7)	0	4 (27)	0	0
Candida	23 (20)	0	0	0	5 (4)	20 (18)	5 (4)	5 (4)	13 (12)	24 (21)	0	18 (16)
Clostridium	1 (11)	0	0	0	1 (11)	1 (11)	0	3 (33)	0	3 (33)	0	0
Coccidioides	0	0	0	0	0	0	0	0	0	2 (100)	0	0
Corynebacterium	2 (5)	0	0	0	2 (5)	10 (27)	2 (5)	0	3 (8)	14 (38)	0	4 (11)
Cryptococcus	4 (50)	2 (25)	0	0	0	1 (13)	0	0	1 (13)	0	0	0
Enterobacter	2 (13)	0	0	0	1 (7)	5 (33)	0	0	0	3 (20)	0	4 (27)
Enterococcus	16 (17)	1 (1)	1 (1)	0	2 (2)	25 (27)	4 (4)	6 (6)	3 (3)	15 (16)	0	20 (22)
E. coli	7 (13)	0	0	0	1 (2)	5 (9)	3 (6)	3 (6)	3 (6)	9 (17)	3 (6)	20 (37)
Klebsiella	2 (5)	0	0	0	1 (3)	9 (24)	0	2 (5)	2 (5)	7 (18)	7 (18)	8 (21)
Mycobacterium tuberculosis	13 (17)	0	2 (3)	1 (1)	5 (7)	17 (22)	2 (3)	7 (9)	6 (8)	21 (28)	0	2 (3)
Pseudomonas	7 (10)	0	0	0	2 (3)	12 (18)	1 (1)	3 (4)	4 (6)	11 (16)	20 (29)	8 (12)
Salmonella	1 (33)	0	0	0	0	0	0	1 (33)	0	1 (33)	0	0
Staphylococcus	28 (19)	0	1 (1)	0	4 (3)	40 (28)	3 (2)	0	0	34 (24)	17 (12)	17 (12)
Stenotrophomonas	4 (33)	0	0	0	0	1 (8)	0	0	1 (8)	1 (8)	5 (42)	0
Streptococcus	19 (28)	0	0	0	2 (3)	13 (19)	1 (1)	2 (3)	2 (3)	13 (19)	3 (4)	13 (19)

*CNS, central nervous system; SST, skin/soft tissue.
